# Hsp90 Is a Novel Target Molecule of CDDO-Me in Inhibiting Proliferation of Ovarian Cancer Cells

**DOI:** 10.1371/journal.pone.0132337

**Published:** 2015-07-02

**Authors:** Dong-Jun Qin, Cai-Xia Tang, Li Yang, Hu Lei, Wei Wei, Ying-Ying Wang, Chun-Min Ma, Feng-Hou Gao, Han-Zhang Xu, Ying-Li Wu

**Affiliations:** 1 Hongqiao International Institute of Medicine, Shanghai Tongren Hospital / Faculty of Basic Medicine, Chemical Biology Division of Shanghai Universities E-Institutes, Key Laboratory of Cell Differentiation and Apoptosis of the Chinese Ministry of Education, Shanghai Jiao Tong University School of Medicine, Shanghai, China; 2 Institute of Oncology, Shanghai 9th People's Hospital, Shanghai Jiao Tong University School of Medicine, 639 Zhi Zao Ju Rd, Shanghai, China; University of Minnesota, UNITED STATES

## Abstract

Synthetic triterpenoid methyl-2-cyano-3, 12-dioxooleana-1, 9(11)-dien-28-oate (CDDO-Me) has been shown as a promising agent against ovarian cancer. However, the underlying mechanism is not well understood. Here, we demonstrate that CDDO-Me directly interacts with Hsp90 in cells by cellular thermal shift assay. CDDO-Me treatment leads to upregulation of Hsp70 and degradation of Hsp90 clients (ErbB2 and Akt), indicating the inhibition of Hsp90 by CDDO-Me in cells. Knockdown of Hsp90 significantly inhibits cell proliferation and enhances the anti-proliferation effect of CDDO-Me in H08910 ovarian cancer cells. Dithiothreitol inhibits the interaction of CDDO-Me with Hsp90 in cells and abrogates CDDO-Me induced upregulation of Hsp70, degradation of Akt and cell proliferation inhibition. This suggests the anti-ovarian cancer effect of CDDO-Me is possibly mediated by the formation of Michael adducts between CDDO-Me and reactive nucleophiles on Hsp90. This study identifies Hsp90 as a novel target protein of CDDO-Me, and provides a novel insight into the mechanism of action of CDDO-Me in ovarian cancer cells.

## Introduction

Ovarian cancer is one of the leading causes of cancer deaths from gynecological malignancy. Despite great advances in chemotherapy and surgical treatment, 70 to 90% of women with ovarian cancer will present a complete response after initial treatment and develop relapse within 2 years and the 5-year survival rate of patients with advanced ovarian cancer remains at approximately 30% [1]. In the USA, estimated 22, 000 new cases of ovarian cancer were predicted to be diagnosed in 2014 resulting in ~14, 000 deaths associated with this disease [2]. Therefore, to improve outcomes for women with advanced ovarian cancer, significant efforts have been devoted to identify protein targeted agents [3].

Heat shock protein 90 (Hsp90) is a highly evolutionarily conserved chaperone protein and is the most well studied member of heat shock protein family. As an ATP-dependent molecular chaperone, Hsp90 plays a critical role in the maturation, stability, and activation of a number of diverse client proteins. Although abundantly expressed in normal cells, its overexpression in malignant cells promotes persistent activation of many cellular kinases and transcription factors from malignancy-induced cellular stresses [4]. Interestingly, many clients or interactors of Hsp90, such as epidermal growth factor receptor (EGFR), human epidermal growth factor receptor 2 (ErbB2), the mammalian target of rapamycin (mTOR) and signal transducer and activator of transcription 3 (STAT3), have been implicated in the pathogenesis of ovarian cancer cells [5–7] and elevated Hsp90 level is common in peritoneal and pleural effusions of patients with advanced–stage ovarian cancer cells [8]. Hsp90 has been considered as an attractive target for ovarian cancer [9–10].

C-28 methyl ester of 2-cyano-3, 12-dioxoolen-1, 9-dien-28-oic acid (CDDO-Me) is a novel synthetic oleanane triterpenoid. CDDO-Me is currently in late-stage clinical development for treatment of chronic kidney disease [11–13] and in phase I/II clinical trials for malignant diseases [14–15]. CDDO-Me exhibits cytotoxicity against a variety of cancer cells including ovarian cancer [16–17], prostate cancer [18] leukemia [19], breast cancer [20], lung cancer [21], pancreatic cancer [22–23] without manifesting any toxicity in normal cells. The mechanistic studies have revealed that CDDO-Me is a multitarget compound. Interestingly, some proteins affected by CDDO-Me such as ErbB2, Akt, STAT3 and mTOR [17] are clients of Hsp90. Therefore, we speculated that Hsp90 might be one target of CDDO-Me, which contributes to the diverse activities of CDDO-Me.

In this study, we demonstrated that Hsp90 is a novel target protein of CDDO-Me in ovarian cancer cells, which contributes to the anti-cancer effect of CDDO-Me in ovarian cancer cells.

## Materials and Methods

### Cell culture

The human epithelial ovarian cancer cells SKOV3 were purchased from the American Type Culture Collection (ATCC, Manassas, VA). HO8910 cell line was obtained from Shanghai Cell Culture Collection (Shanghai, China). HO8910 cell line was cultured in RPMI-1640 (Gibco, Foster City, CA) supplemented with 10% (w/v) fetal bovine serum (FBS; Gibco) and 1% penicillin-streptomycin (Gibco). SKOV3 cell line was cultured in McCoy’s 5A (Gibco, Foster City, CA) supplemented with 10% (w/v) fetal bovine serum (FBS; Gibco) and 1% penicillin-streptomycin (Gibco). All cell lines were maintained at 37°C in a humidified atmosphere with 5% CO_2_.

### Western Blotting

Cells were washed with PBS and lysed with lysis buffer (50 mM Tris-HCl, pH 6.8, 100 mM DTT, 2% SDS, 10% glycerol). Cell lysates were centrifugated at 20,000g for 10 min, and proteins in the supernatants were quantified. Protein extracts were equally loaded to 8% to 12% SDS–polyacrylamide gel, electrophoresed, and transferred to nitrocellulose membrane (Bio-Rad). The blots were stained with 0.2% Ponceau S red to ensure equal protein loading. After blocking with 5% nonfat milk in PBS, the membranes were probed with antibodies against Hsp90(Santa Cruz Biotech, Santa Cruz, CA), Hsp70 (Santa Cruz Biotech), vinculin (Santa Cruz Biotech), Akt (Santa Cruz Biotech), ErbB2 (Cell Signaling, Beverly, MA), Erk (Cell Signaling), phosphorylated Erk (Cell Signaling). The signals were detected with a chemiluminescence phototope-HRP kit (Cell Signaling) according to manufacturer’s instructions. As necessary, blots were stripped and reprobed with anti-β-actin antibody as internal control. All experiments were repeated three times with similar results.

### Cellular Thermal Shift Assay (CETSA)

PBS diluted HO8910 cell suspensions were freeze-thawed three times with liquid nitrogen. The soluble fraction (lysate) was separated from the cell debris by centrifugation at 20, 000 × g for 20 min at 4°C. The cell lysates were diluted with PBS and divided into two aliquots, with one aliquot treated with DMSO and the other aliquot with CDDO-Me (25 μM). After 10–30 min incubation at room temperature the respective lysates were divided into smaller aliquots (30 μL) and heated individually at different temperatures for 3 min (Veriti thermal cycler, Applied Biosystems/Life Technologies) followed by cooling for 3 min at room temperature. The appropriate temperatures were determined in preliminary CETSA experiments (data not shown). The heated lysates were centrifuged at 20, 000 × g for 20 min at 4°C in order to separate the soluble fractions from precipitates. The supernatants were transferred to new microtubes and analyzed by sodium dodecyl sulfate polyacrylamide gel electrophoresis (SDS-PAGE) followed by western blot analysis. Dose effect of CDDO-Me on the stability of Hsp90 was evaluated similarly.

To examine the effect of DTT on the binding of CDDO-Me to Hsp90 in living cells, HO-8910 cells were treated with DMSO, DTT (2 mM), CDDO-Me (25 μM), or CDDO-Me (25 μM) preincubated with DTT (2 mM, 30 min) for 3 hours. Then, cells were harvested and diluted in PBS supplemented with complete protease inhibitor cocktail and heated at 63°C for 3 min, followed by cooling at room temperature for another 3 min. The cell suspensions were freeze-thawed two times with liquid nitrogen. The lysates were centrifuged at 20, 000 × g for 20 min at 4°C in order to separate the soluble fractions from precipitates. The supernatants were transferred to new microtubes and analyzed by sodium dodecyl sulfate polyacrylamide gel electrophoresis (SDS-PAGE) followed by western blot analysis.

### RNA Interference and Transfection

Short interfering RNA (siRNA) targeting human Hsp90 were synthesized by Biomics Biotechnologies, Jiangsu, China. The targeting sequences are as follows, 5’-GUAUUGUCACAAGCACAUAdTdT-3’ and 5’-CGUCUCGCAUGGAAGAAGU-3’. Universal negative control siRNA (5’-UUCUCCGAACGUGUCACGUdTdT-3’) was used as a control. HO-8910 cells were seeded (3×10^5^ cells/1500 μl/well of a 6-well plate), and then mixed with lipofectamine-RNA complex solution [100 nM RNA, 5 μl lipo2000, 500 μl Opti-MEM/well], according to manufacturer's recommendation. Forty eight hours after transfection of siRNAs, cells were subjected to the immunoblotting assay or other experiments.

### Cell Proliferation Assay

HO-8910 or SKOV3 cells were treated with CDDO-Me alone or in combination with DTT. Cell proliferation was determined using a Cell Counting Kit (Laboratories, Kumamoto, Japan) according to the manufacturer’s instructions.

### Statistical analysis

Student’s t-test was used to evaluate the difference between two different treatments. A *p* value of less than 0.05 was considered statistically significant.

## Results

### CDDO-Me interacts with Hsp90 in cells

**Fig 1:**
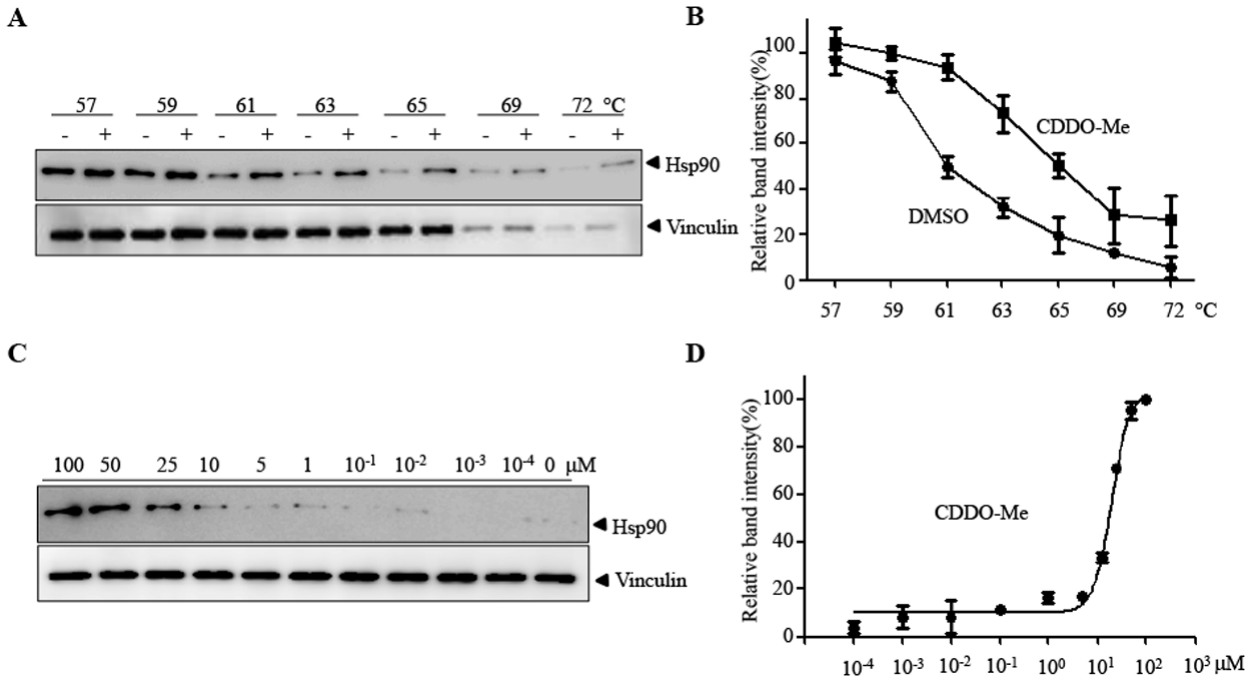
CDDO-Me interacts with Hsp90 in cells. CETSA was performed on HO8910 cells as described in materials and methods. The stabilization effect of CDDO-Me on Hsp90 and vinculin at different temperatures (A) and different doses (C) were evaluated by western blot. The intensity of the Hsp90 bands was quantified by quantity one software (B, D). Each experiment was repeated as least three times.

In order to know whether CDDO-Me interacts with Hsp90 in cells, CETSA, a newly developed method to evaluate drug binding to target protein in cells, was performed. Ovarian cancer cells HO8910 were lysed and incubated with DMSO or CDDO-Me for half an hour at room temperature. Then, multiple aliquots of cell lysate were heated to different temperatures. After cooling, the samples were centrifugated to separate the soluble fractions from precipitated proteins and the presence of Hsp90 in the soluble fraction was examined by western blot. As shown in Fig 1A and 1B, compared with DMSO, the presence of CDDO-Me markedly increased the accumulation of Hsp90 in the soluble fraction at the temperatures examined. We also test the dose-response of CDDO-Me on Hsp90 stability to heating. As shown in Fig 1C and 1D, with the increase of CDDO-Me concentration, the accumulation of Hsp90 markedly increased. As a negative control, CDDO-Me could not increase the stability of vinculin in cells. These data suggest that CDDO-Me directly interacts with Hsp90 in cells.

### CDDO-Me inhibits Hsp90 activity in cells

**Fig 2:**
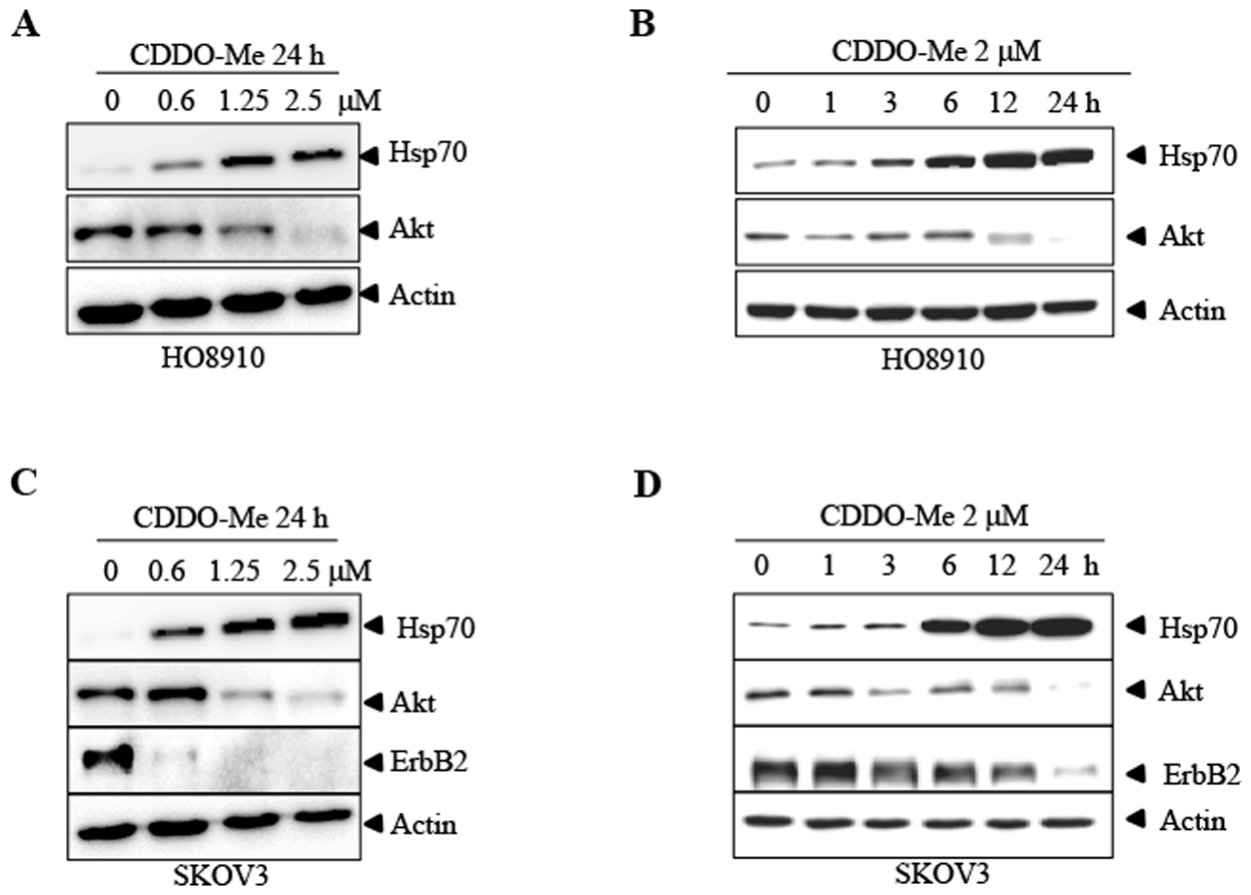
CDDO-Me inhibits Hsp90 function in cells. A-D, HO8910 and SKOV3 cells were treated with CDDO-Me at different dose (A, C) and different time (B, D), and the indicated proteins were detected by western blot. Each experiment was repeated as least three times.

One of the hallmarks of Hsp90 inhibition is the induction of Hsp70 and this response has been used as a biomarker for many Hsp90 inhibitor clinical trials [24]. If CDDO-Me inhibits Hsp90, it might upregulate the expression of Hsp70. Indeed, CDDO-Me treatment leads to the upregulation of Hsp70 in a dose and time dependent manner (Fig 2A and 2B), which is similar to that observed in STA-9090, a known Hsp90 inhibitor, treated SKOV3 and HO8910 cells (S1 Fig). Another hallmark of Hsp90 inhibition is the degradation of client proteins [25]. Some of them, like ErbB2 have been reported to be very sensitive to Hsp90 inhibition, whereas others like Akt are reported to be less sensitive [26–27]. We examined the protein level of ErbB2 and Akt in CDDO-Me treated cells. Expression of ErbB2 is detectable in SKOV3 cells but not in H08910 cells (data not shown). CDDO-Me treatment leads to the decrease of ErbB2 protein in a dose and time dependent manner in SKOV3 cells (Fig 2C and 2D). For the Akt protein, CDDO-Me treatment leads to the decrease of Akt in a dose and time dependent manner in both HO8910 cells (Fig 2A and 2B) and SKOV3 cells (Fig 2C and 2D). These data suggest that CDDO-Me inhibits Hsp90 activity in ovarian cancer cells.

### Knockdown of Hsp90 on the effect of CDDO-Me on ovarian cancer cells

**Fig 3:**
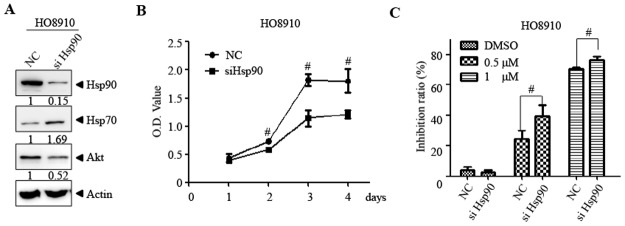
Knockdown of Hsp90 inhibits cell proliferation and enhances the anticancer effect of CDDO-Me in HO8910 cells. HO8910 cells were transfected with control siRNA (HO8910^siNC^) or Hsp90 specific siRNA (HO8910^siHsp90^), then treated with or without CDDO-Me for indicated times. The indicated proteins were detected by western blot and the intensity of the bands was quantified by quantity one software (A). Cell proliferation was examined by CCK-8 assay (B, C). Each experiment was repeated as least three times. ^#^ p<0.05, compared to control siRNA transfected cells.

To determine whether inhibition of Hsp90 correlated with CDDO-Me induced inhibition of cell proliferation, we used RNA interference to reduce Hsp90 expression in HO8910 cells. As shown in Fig 3A, Hsp90 protein was specifically reduced by Hsp90-targeting siRNA (HO8910^siHsp90^) compared to the non-specific siRNA (HO8910^siNC^). Compared with the vector transfected cells, knockdown of Hsp90 significantly inhibited cell growth (Fig 3B) and enhanced CDDO-Me induced proliferation inhibition (Fig 3C) in HO8910 cells. These data suggest that Hsp90 contributes to CDDO-Me induced proliferation inhibition in ovarian cancer cell.

### The interaction of CDDO-Me with Hsp90 in ovarian cancer cells by its α,β-unsaturated carbonyl groups on rings A and C

**Fig 4:**
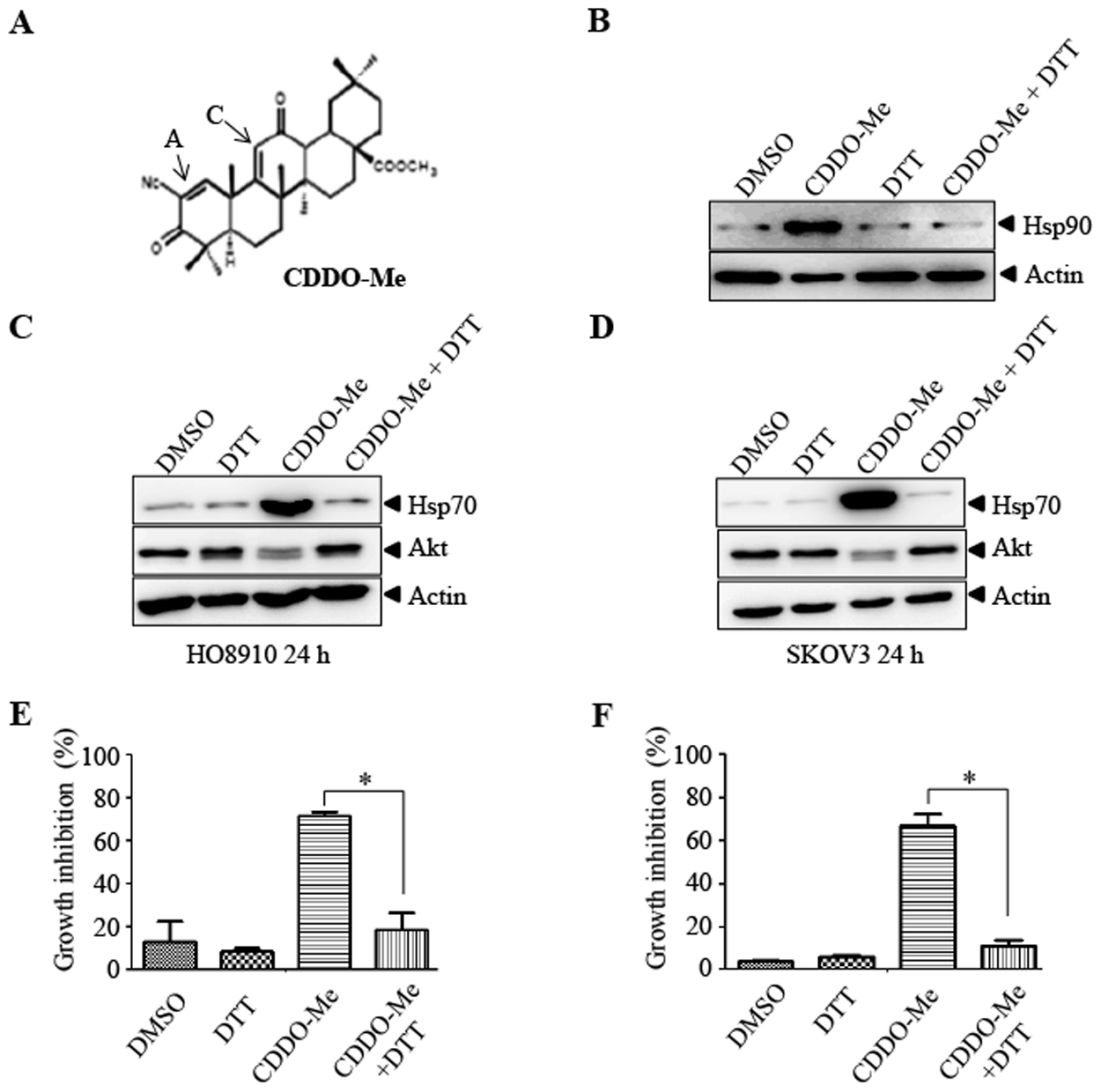
DTT inhibits the interaction of Hsp90 with CDDO-Me in cells. A. Chemical structure of CDDO-Me. B. Living HO8910 cells treated with DTT, DMSO, CDDO-Me (25 μM) and CDDO-Me (25 μM) preincubated with DTT (2 mM), then were subject to CETSA as described in material and methods. The stability changes of Hsp90 were evaluated by western blot (B). HO8910 and SKOV3 cells were treated with CDDO-Me (2 μM) for 24 hours in the presence or absence of DTT (2 mM). The indicated proteins were detected by western blot (C, D) and cell viability was examined by CCK-8 assay (E, F). Each experiment was repeated as least three times. * p<0.05.

Structure-activity analysis has shown that the α,β-unsaturated carbonyl groups on rings A and C is key to the activity of CDDO-Me (Fig 4A) [11]. DTT could form reversible adducts with CDDO-Me α,β-unsaturated carbonyl groups and abrogate its activity. To test whether the interaction of CDDO-Me with Hsp90 in living cells depends on the α,β-unsaturated carbonyl moiety, CDDO-Me (25 μM) preincubated with or without DTT (2 mM) was used to treat HO8910 cells, followed with CETSA examination. Indeed, DTT could abrogated the stabilizing effect of CDDO-Me on Hsp90 (Fig 4B). Consistent with this, DTT treatment completely inhibited CDDO-Me (2 μM) induced increase of Hsp70 (Fig 4C and 4D) and inhibition of cell proliferation (Fig 4E and 4F). DTT has been widely used as antioxidant to reduce the ROS level in cells and CDDO-Me has also been shown to increase the ROS level in cells. Thus, DTT may inhibit Hsp70 expression by reducing ROS. However, H_2_O_2_ treatment did not upregulate the expression of Hsp70 (S2 Fig). These data suggest that CDDO-Me may interact with Hsp90 through its α,β-unsaturated carbonyl groups.

## Discussion

CDDO-Me has shown great efficacy against a variety of cancers, including ovarian cancer. However, the underlying mechanism of action of CDDO-Me on ovarian cancer cells is less clear. In this study, we demonstrated that Hsp90 is a novel target protein of CDDO-Me and targeting Hsp90 contributes to CDDO-Me induced cell death of ovarian cancer cells.

Intensive studies on the action of CDDO-Me have revealed a lot target proteins including Akt, Nrf2, PTEN, ErbB2, IKKβ, STAT3, mTOR, PPARγ et.al [12]. Interestingly, some of them such as ErbB2, STAT3 and Akt are substrates of Hsp90, we therefore postulate that CDDO-Me may directly inhibit Hsp90, which in turn mediates the multiple effect of CDDO-Me. To test this hypothesis, we used the CETSA method to evaluate the interaction between CDDO-Me and Hsp90. CETSA utilizes the concept that target proteins usually get stabilized when drug molecules bind. Compared with other methods to confirm the interaction of small molecule compounds with proteins, CETSA is more feasible to perform and can directly measure whether a drug molecule reach its targets in cells and animal models [28–29]. Using this method, we demonstrated that CDDO-Me interacts with Hsp90 in cells. This was further supported by the induction of Hsp70, a universe response observed in cells upon treated with Hsp90 inhibitors, especially those targeting the N-terminus of Hsp90 [30]. Moreover, the client proteins of Hsp90, such as ErbB2 and Akt, could also be reduced by CDDO-Me treatment. Therefore, we propose that CDDO-Me interacts with Hsp90 and inhibits its activity in cells.

One important question is whether the interaction of CDDO-Me with Hsp90 contributes to the anticancer effect of CDDO-Me on ovarian cancer cells. Similar to that observed in CDDO-Me treated cell, specifically silencing of Hsp90 results in the induction of Hsp70 and down-regulation of Akt in HO8910 cells (Fig 3A) [31–32]. Moreover, knockdown of Hsp90 could significantly enhance the proliferation inhibition effect of CDDO-Me in HO8910 cells. These data support that targeting Hsp90 contributes the anti-ovarian cancer effect of CDDO-Me. As CDDO-Me is a known multiple targets compound, we don’t rule out that other target proteins also contribute to the anti-ovarian cancer effect of CDDO-Me.

CDDO-Me has two α,β-unsaturated carbonyl moiety. The proposed mechanism underlying the anti-cancer effect of CDDO-Me is by the formation of Michael adducts between CDDO-Me and reactive nucleophiles, such as free thiols on target proteins [33]. CDDO-Me may interact with Hsp90 in a similar way, because DTT, a –SH group containing compound, abrogates CDDO-Me-induced stabilization of Hsp90 in response to heating. Moreover, CDDO-Me induced up-regulation of Hsp70 and reduction of Akt could also be reversed by DTT in cells. As CDDO-Me could increase the ROS level in cancer cells and inhibiting ROS blocks the effect of CDDO-Me [23], one may argue that the effect of DTT on Hsp70 may be due to its antioxidant activity. However, increasing ROS by H_2_O_2_ could not upregulate the expression of Hsp70 (S2 Fig). Thus, DTT most likely reverses the effect of CDDO-Me by directly interacting with CDDO-Me. The binding site of CDDO-Me on Hsp90 is currently not known. There are 6 cysteines in Hsp90 protein. Which cysteine(s) contribute to the interaction of CDDO-Me with Hsp90 warrants further investigation.

To our knowledge, this is the first report to show that Hsp90 is a novel target protein of CDDO-Me. Targeting Hsp90 provides a novel explanation for the multiple effects of CDDO-Me observed in different cancer cells. The application of CDDO-Me to cancer cells with Hsp90 overexpression deserves further preclinical and clinical investigations.

## Supporting Information

S1 Fig STA-9090 inhibits Hsp90 function in cells. HO8910 (A) and SKOV3 (B) cells were treated with STA-9090 for different times and the indicated proteins were detected by western blot. Each experiment was repeated as least three times.
(TIF)

S2 Fig H_2_O_2_ activates Erk, but does not upregulate Hsp70. HO8910 (A) and SKOV3 (B) cells were treated with H_2_O_2_ (10 μM) for different times and the indicated proteins were detected by western blot. Each experiment was repeated as least three times.
(TIF)
