# Supplementary figures and images for: Hsp90 Is a Novel Target Molecule of CDDO-Me in Inhibiting Proliferation of Ovarian Cancer Cells
Source: PLoS One. 2015 Jul 2;10(7):e0132337. doi: 10.1371/journal.pone.0132337 (PMC4489813; doi:10.1371/journal.pone.0132337)

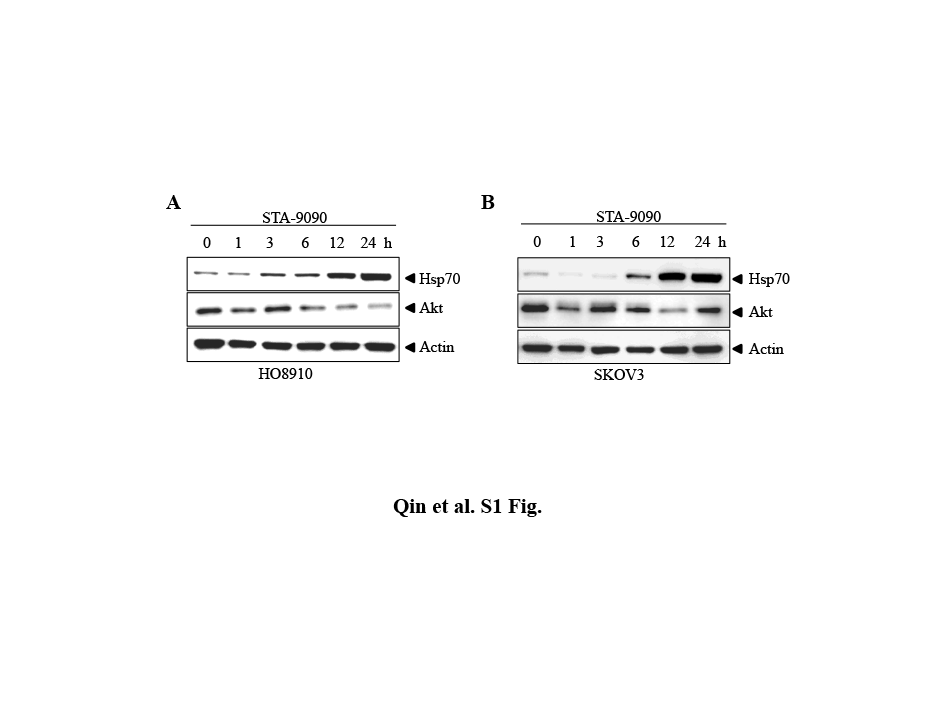

Supplement: S1 Fig — HO8910 (A) and SKOV3 (B) cells were treated with STA-9090 for different times and the indicated proteins were detected by western blot. Each experiment was repeated as least three times. (TIF) [file pone.0132337.s001.tif]

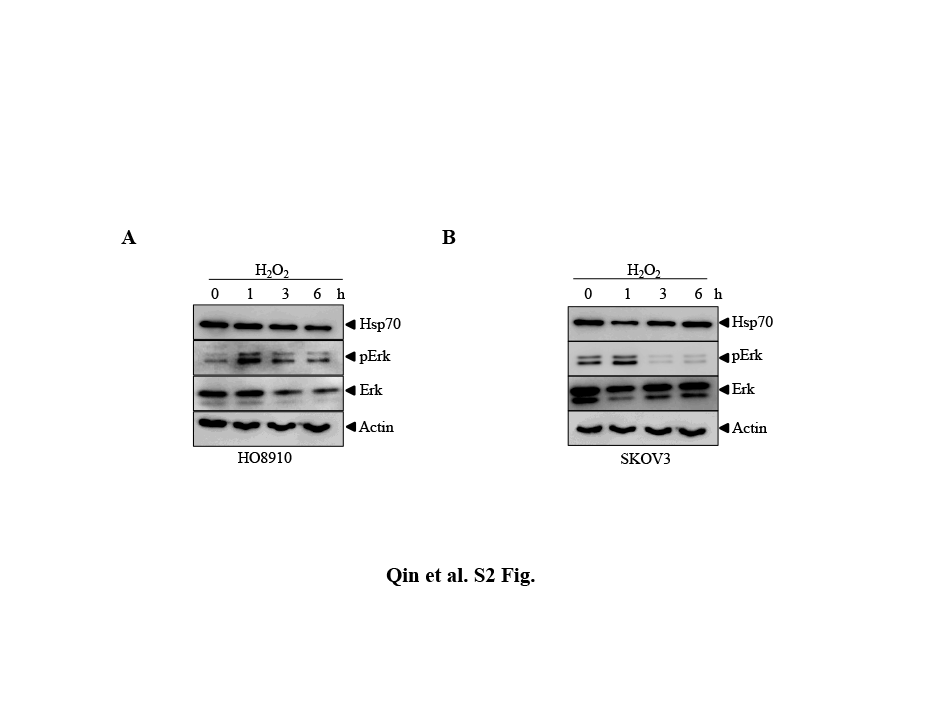

Supplement: S2 Fig — HO8910 (A) and SKOV3 (B) cells were treated with H2O2 (10 μM) for different times and the indicated proteins were detected by western blot. Each experiment was repeated as least three times. (TIF) [file pone.0132337.s002.tif]
